# The fluorescence patterns of antinuclear antibodies: morphological characteristics, immunological correlations and clinical significance

**DOI:** 10.3389/fimmu.2026.1979009

**Published:** 2026-10-08

**Authors:** Yusi Liu, Xu Han, Xiuying Shi

**Affiliations:** 1 National Clinical Research Center for Medical Auxiliary Technology(Laboratory Medicine), Department of Laboratory Medicine, The First Hospital of China Medical University, Shenyang, China; 2 Department of Pathology, The First Hospital of China Medical University, Shenyang, China

**Keywords:** antinuclear antibodies, clinical significance, fluorescence patterns, immunological correlations, morphological characteristics

## Abstract

Most institutions adhere to the recommendations of the International Consensus on Antinuclear Antibody (ANA) Patterns (ICAP) for the nomenclature of ANA patterns and the standardization of laboratory reports. Some ANA patterns are frequently encountered in routine clinical laboratories, whereas others are rare. Additionally, certain patterns share highly similar morphological features and are therefore difficult to differentiate. Most ANA patterns are of high clinical relevance to the diagnosis of autoimmune diseases, while the clinical significance of certain patterns remains undefined. Each staining pattern corresponds to specific immunological epitopes. For different antigen targets within the same pattern, their clinical relevance may be partially comparable yet distinctly different in other aspects. Accurate identification of ANA patterns and comprehensive understanding of the clinical correlations among different patterns, as well as among distinct antigen targets of the same pattern, are of critical importance. Nevertheless, it remains challenging for laboratory analysts to distinguish subtle textural variations and other morphological characteristics of immunofluorescence staining patterns under light microscopy. Based on recent advances in relevant research, this review highlights the characteristic distinctions of morphologically similar ANA patterns, their immunological target antigens, and the similarities and differences in clinical relevance among various targets within an identical pattern. This review aims to assist laboratory professionals and clinicians in better identifying and interpreting ANA staining patterns, thereby optimizing the clinical application of HEp-2 indirect immunofluorescence (IFA) assays in the diagnostic workup of patients with suspected autoimmune diseases.

## 1 Introduction

Antinuclear antibodies (ANAs) represent a crucial category of autoantibodies widely used for the screening of autoimmune diseases. ANAs can be assayed via multiple approaches, including indirect immunofluorescence assay (IFA), immunoblotting assay, chemiluminescence assay, etc (1). Among these methods, HEp-2 IFA is currently the most widely adopted technique for ANA detection, as it identifies the broadest spectrum of immunofluorescence patterns and is capable of recognizing uncharacterized autoantibody targets (2, 3). Relevant clinical guidelines recommend HEp-2 IFA as the first-line screening assay for ANA testing, with additional confirmatory methods subsequently applied to define specific antigenic targets (2, 3). HEp-2 IFA yields three core diagnostic parameters: ANA positivity, immunofluorescence pattern, and endpoint titer (2, 3). Pattern types and titers help differentiate individuals without autoimmune diseases from patients with suspected autoimmune disorders. Furthermore, HEp-2 IFA staining patterns offer preliminary clues regarding potential autoantibody specificities, enabling targeted detection of the most probable disease-related autoantibodies and thereby supporting clinical diagnosis and therapeutic decision-making.

The International Consensus on ANA Patterns (ICAP) classifies HEp-2 IFA patterns into 32 distinct patterns (AC-0 to AC-31) and AC-XX (encompassing certain special and unclassified patterns) (4, 5). These patterns are categorized into five main groups: negative (n=1), nuclear (n=17), cytoplasmic (n=9), mitotic (n=5), and unclassified patterns (with an unknown number) (4, 5). While some patterns are easily distinguishable, others are similar and thus difficult to differentiate. However, recognizing subtle differences in staining texture and other features can be challenging for analysts examining samples under the microscope.

We classified and compared AC1-AC31 based on the similarity of their fluorescence patterns. Each pattern corresponds to specific immunological epitopes, and most antinuclear antibodies are highly relevant to the diagnosis of autoimmune diseases, although the clinical significance of some patterns remains unclear. Since each pattern is associated with distinct autoantibody specificities and clinical contexts, the accurate identification of these patterns becomes crucial. Based on recent studies, this review aims to summarize and consolidate the characteristics of each pattern, their immunological targets, and the similarities and differences in clinical relevance among targets within the same pattern. The focus is placed on pattern morphology and texture, associations with autoantibodies, and clinical relevance to autoimmune diseases.

## 2 Methods for antinuclear antibody testing

After initial screening by IFA using HEp-2 cells and/or liver cell lines, second-line confirmatory testing—including enzyme-linked immunosorbent assay (ELISA), immunoblotting assay, and radioimmunoassay (RIA), either qualitative or semi-quantitative—should be performed for the precise detection of specific antinuclear antibodies (1, 6). RIA is restricted to laboratories with dedicated facilities for radioisotope handling (1, 6). Following the identification of specific antibody profiles, serial monitoring of antibody levels can be implemented via chemiluminescence immunoassay or ELISA (1, 6). This strategy aids clinicians in evaluating disease diagnosis, therapeutic response and clinical prognosis.

## 3 Nuclear patterns of HEp-2 IFA: fluorescence pattern characteristics, immunological epitopes and clinical relevance

Nuclear patterns are classified according to similarity into: nuclear patterns with stained metaphase chromosome plate [homogeneous (AC-1), dense fine speckled (AC-2), Topo I-like (AC-29), fine-speckled with mitotic plate (AC-30)] (7), centromere and discrete nuclear dots patterns [centromere/CENP (AC-3), multiple nuclear dots (AC-6), few nuclear dots (AC-7)], nuclear speckled patterns [fine speckled (AC-4), large/coarse speckled (AC-5), myriad discrete speckled (AC-31)], nucleolar patterns [homogeneous nucleolar (AC-8), clumpy nucleolar (AC-9), punctate nucleolar (AC-10)], nuclear membrane patterns [smooth nuclear envelope (AC-11), punctate nuclear envelope (AC-12)], nuclear polymorphic patterns [Anti-PCNA (AC-13) and CENP-F-like (AC-14)] (2). The morphological characteristics, target antigens and clinical relevance of nuclear patterns are shown in **Table 1**.

**Table 1:** The morphological characteristics, target antigens and clinical relevance of nuclear patterns.

Nuclear patterns		Morphological characteristics	Antigens	Disease association
Nuclear patterns with stained metaphase chromosome plate	AC-1	The homogeneous fluorescence in nuclei of interphase cell and chromosomes of mitotic cells	dsDNA	SLE (diagnostic criteria)
			Histones, Nucleosomes	SLE, DIL
			Unknown antigens	RA, AIH, Juvenile Idiopathic Arthritis, other conditions
	AC-2	The speckled fluorescence of heterogeneous size, intensity, and distribution in the nuclei of interphase cells and chromosomes of mitotic cells	DFS70	Non-autoimmune diseases, Healthy individuals, SARD (rare)
	AC-29	The combination of five characteristic features	Topo I (Scl-70)	SSc (classification criteria), SLE and other SARD (rare)
	AC-30	The evenly distributed, fine speckled fluorescence throughout the interphase nucleus and the metaphase chromosome plate	The coexistence of multiple autoantibodies	Systemic autoimmune rheumatic disease
			Unknown antigens	Individuals without overt autoimmune diseases
Centromere and discrete nuclear dots patterns	AC-3	The discrete, scattered, coarse speckled fluorescence in the nuclei of interphase cells andchromosomes of mitotic cells (typically 40–80/cell)	CENP-A, CENP-B, CENP-C	SSc (diagnostic criteria), SjD, PBC, SLE, other conditions.
	AC-6	The discrete, bright, spot-like fluorescence in the nuclei of interphase cells (typically 6–20/cell)	SP100, PML, SP140	PBC (common type), PSC, AIH, SLE, Hepatitis C, other conditions
			NXP-2	DM (JDM and adult DM)
	AC-7	A small number of discrete dot-like fluorescent structure in the nuclei of interphase cells (typically 1–6/cell)	p80-coilin and SMN complex	Low disease positive predictive value
Nuclear speckled patterns	AC-4	The fine speckled fluorescence in the interphase nucleus and the nuclear areas outside the chromosomes of mitotic cells	SSB/La	SjD (common type), SLE, other conditions
			Mi-2, T1F1γ	Myositis-specific antibodies (MSA)
			TIF1β	Tumor-associated myositis
			Ku	Myositis-related antibodies (MAA), SSc-AIM, SLE-SSc-AIM overlap
			TRIM21/Ro52 (uncertain)	SjD, SLE, etc.
	AC-31	The discrete scattered granules in the interphase nucleus and the nuclear areas outside the chromosomes of mitotic cells	TROVE2/Ro60	SjD (diagnostic criteria), SLE, Healthy individuals (rare)
	AC-5	The coarse speckled fluorescence in the interphase nucleus and the nuclear areas outside the chromosomes of mitotic cells	Sm	SLE (diagnostic criteria)
			U1RNP	MCTD (diagnostic criteria)
			hnRNP	RA (hnRNPA2/B1), SSc(hnRNPA1, B1), Unknown (hnRNP C1, C2)
			RNA polymerase III	SSc, particularly dcSSc
Nucleolar patterns	AC-8	The diffuse homogeneous fluorescence staining of the entire nucleolus in interphase cells	PM/Scl-75, PM/Scl-100	PM/SSc, DM, dcSSc
			Th/To	SSc, particularly lcSSc
			C23/nucleolin, B23/nucleophosmin, NO55/SC65	Remains unclear
	AC-9	The irregular clumpy staining of the nucleoli and Cajal bodies in interphase cells and perichromosomal staining surrounding the metaphase plate	U3-snoRNP/fibrillarin	SSc, particularly dcSSc
	AC-10	The dense, distinct granular staining confined to the nucleoli of interphase cells and one to five pairs of bright nucleolar organizer regions (NORs) within the chromatin in metaphase cells	RNApol-I	SSc, particularly dcSSc
			hUBF/NOR-90	SSc, SjD, RA, SLE, UCTD
Nuclear membrane patterns	AC-11	The homogeneous nuclear staining with intensified fluorescence at the nuclear periphery	Lamins A, Lamins B, Lamins C, LAP	No disease specificity
	AC-12	The punctate staining of the nuclear envelope in interphase cells and enhanced fluorescence at the contact sites between adjacent cells	GP210, p62, LBR	PBC
			Tpr	No disease specificity
Nuclear polymorphic patterns	AC-13	The multiple rows of nuclear speckles with variable size and fluorescence intensity	PCNA	SLE (controversial), other conditions
	AC-14	The numerous regularly arranged, small, and faint fluorescent dots staining of centromeres in prometaphase and metaphase cells	CENP-F	Malignancies, autoimmune and neurological diseases

SLE, systemic lupus erythematosus; DIL, drug-induced lupus; RA:rheumatoid arthritis; AIH:autoimmune hepatitis; SARDs, systemic autoimmune rheumatic diseases; SSc systemic sclerosis; dcSSc, diffuse cutaneous systemic sclerosis; lcSSc, limited cutaneous systemic sclerosis; SjD, Sjögren disease; PBC, primary biliary cholangitis; AIM, autoimmune myopathy; MCTD:mixed connective tissue disease; DM, dermatomyositis; PM:polymyositis; JDM, juvenile dermatomyositis; UCTD, undifferentiated connective tissue disease.

### 3.1 Nuclear patterns with stained metaphase chromosome plate

The nuclear homogeneous pattern (AC-1) is characterized by positive staining of interphase cell nuclei with homogeneous fluorescence (2, 7, 8). Nucleoli may stain negative or positive depending on the cell substrate used (2, 7, 8). In mitotic cells, chromosomes exhibit intense, concentrated homogeneous fluorescence, while the peri-chromosomal fluorescence signal is weaker (2, 7, 8). Target antigens associated with the AC-1 pattern include double-stranded DNA (dsDNA), histones, nucleosomes, and other uncharacterized nuclear antigens (2, 8). When a patient is positive for anti-dsDNA, anti-histone, and anti-nucleosome antibodies, systemic lupus erythematosus (SLE) and drug-induced lupus (DIL) should be considered in diagnosis (2, 7, 9). Anti-dsDNA antibody is highly specific for SLE and, together with anti-Sm antibody, is incorporated into the SLE diagnostic criteria (10, 11). It also plays a critical role in monitoring SLE disease activity and assessing therapeutic response (10, 11). Anti-histone and anti-nucleosome antibodies are frequently detected in patients with SLE and DIL, often concurrently with anti-dsDNA antibody (7, 12, 13). Anti-nucleosome antibody demonstrates high diagnostic accuracy for differentiating between autoimmune hepatitis (AIH) and DIL (7, 12). Additionally, these two antibodies can be detected in other connective tissue diseases and show a negative correlation with pulmonary fibrosis complications in systemic sclerosis (SSc) and mixed connective tissue disease (MCTD) (7, 13–15). When the AC-1 pattern is present alongside negative results for dsDNA, histone, and nucleosome antibodies, underlying conditions such as rheumatoid arthritis (RA), AIH, or juvenile idiopathic arthritis (JIA) should be considered (2, 7, 9). In some cases, strong positivity for condensed chromatin may be associated with negative results for anti-dsDNA antibodies. It is worth noting, however, that some recent studies have also highlighted the opposite situation, namely the detection of anti-dsDNA antibodies by CLIA, FEIA, or immunoblot assays in patients with a negative ANA pattern (16–18). This finding might be related to differences in the affinity of the individual methods for specific epitopes of these autoantigens (18).

The nuclear dense fine speckled pattern (AC-2) is characterized by positive staining within the nuclei of interphase cells, manifesting as speckled fluorescence of heterogeneous size, intensity, and distribution (2, 7, 8). This pattern creates alternating sparse and dense intranuclear regions, resulting in discontinuous and irregular nuclear margins (2, 7, 8). In mitotic cells, the pattern is evident on condensed chromosomes, which display intensified speckled fluorescence with uneven size, brightness, and distribution, while the surrounding chromosome regions remain fluorescence-negative (2, 7, 8). The AC-2 pattern targets a single major autoantigen, DFS70 (2, 7, 8, 19). AC-2 is most frequently associated with isolated anti-DFS70 antibodies, as the coexistence of additional antibodies to nuclear antigens usually disturbs the genuine characteristics of the AC-2 pattern, frequently rendering an AC-30 pattern or a mixed nuclear pattern. Anti-DFS70 antibodies are frequently detected in healthy individuals or in patients without systemic autoimmune rheumatic diseases (SARDs) (2, 5, 7, 9, 20). Due to its high negative predictive value for SARDs (5, 20), the accurate identification of the AC-2 pattern is of critical clinical importance.

The Topo I-like pattern (AC-29) is defined by five characteristic features (21) (1). Interphase cell nuclei exhibit a fine speckled fluorescence pattern (AC-4) (2). Condensed chromosomes in mitotic cells display intensified fine speckled fluorescence. However, at high antibody titers, the chromosomal fluorescence may appear more homogeneous (3). The nucleolar organizer regions (NORs) of mitotic cells show strong staining. This NOR staining can sometimes be obscured by intense chromosomal fluorescence, as NORs may not always lie within the same focal plane (4). The cytoplasm of interphase and mitotic cells presents weak, faint, mesh-like fluorescence radiating from the perinuclear region. This cytoplasmic staining may become more distinct at higher serum dilutions (5). Interphase cells may exhibit variable nucleolar-associated staining, which can appear as punctate or perinucleolar patterns. This feature is not obligatory and is not a universal hallmark AC-29 pattern. The AC-29 pattern is strongly associated with antibodies against DNA topoisomerase I (Topo-I) (22). The target antigen for anti-Scl-70 antibodies is Topo-I, a relatively specific serological marker for systemic sclerosis (SSc) (7, 22, 23). Consequently, the AC-29 pattern is strongly associated with SSc, particularly the diffuse cutaneous subtype and more aggressive forms of the disease (7, 22, 23). Beyond SSc, anti-Topo-I antibodies are linked to progressive renal injury and fibrosis affecting the lungs, musculoskeletal system, and heart (7, 24). Anti-Scl-70 antibodies have also been identified in a subset of patients with SLE (7, 25, 26). Studies report that 96.2% of SSc patients exhibiting the AC-29 pattern are positive for anti-Scl-70 antibodies (27). However, rare SSc cases may display the Topo-I nuclear pattern while remaining seronegative for anti-Scl-70 antibodies (28).

The fine-speckled mitotic plate pattern (AC-30) is characterized by evenly distributed, fine speckled fluorescence throughout the interphase nucleus, with speckles of consistent size, intensity, and distribution (5, 7). A similar speckled staining pattern is also observed on the metaphase chromosome plate (5, 7). Differentiation from mixed nuclear fluorescence patterns is essential. For instance, the concurrent presence of a nuclear homogeneous pattern (AC-1) and a nuclear speckled subtype results in a uniformly stained (homogeneous) metaphase plate alongside speckled nuclear fluorescence. Such cases should be categorized as a mixed pattern rather than AC-30. AC-30-positive specimens are associated with unknown autoantibodies or the coexistence of multiple clinically relevant autoantibodies (anti-dsDNA, nucleosome, Sm, U1-RNP, topo-I, TROVE2/Ro60, etc) (5, 7, 19). The coexistence of these autoantibodies can mask the expression of their respective characteristic immunofluorescence patterns, giving rise to the AC-30 phenotype. Accordingly, AC-30 can be regarded as a specific subtype of a mixed pattern (5, 7). In addition, some AC-30-positive samples contain anti-DFS70 antibodies in combination with other antinuclear antibodies (ANAs), which may obscure the classic AC-2 pattern typically associated with anti-DFS70 reactivity (7, 19). The AC-30 fluorescence pattern is most frequently detected in individuals without overt autoimmune disease (5, 7). Nevertheless, as this pattern may represent the combined reactivity of multiple ANAs, it can occasionally be identified in patients with systemic autoimmune rheumatic diseases, particularly those with multiple ANA specificities (5, 7).

### 3.2 Centromere and discrete nuclear dots patterns

The centromere/CENP pattern (AC-3) is characterized by discrete, scattered, coarse speckled fluorescence within the nuclei of interphase cells, typically numbering 40–80 per cell with varying sizes (2, 8). In mitotic cells, this pattern manifests as one (in metaphase) or two (in anaphase) parallel bands of condensed speckled fluorescence on the chromosomes (2, 8). The target antigens for the AC-3 pattern are primarily CENP-A, B, and C (2, 8). This pattern is highly associated with SSc, particularly the limited cutaneous form (lcSSc) (8, 9, 29). Consequently, anti-centromere protein antibodies are included in the diagnostic criteria for SSc (9, 29). Furthermore, the AC-3 pattern is observed in patients with primary biliary cholangitis (PBC) or PBC-SSc overlap syndrome (2, 8, 9, 30). In Sjögren Disease (SjD), antibodies against both CENP-B and CENP-C may coexist, or antibodies specific only to CENP-C may be present (31).

The multiple nuclear dots pattern (AC-6) is characterized by discrete, bright, spot-like fluorescence of varying size, number, and intensity distributed within the nuclei of interphase cells (typically 6–20 dots) (2, 8). Cells in mitosis exhibit negative chromosomal staining, although dot-like fluorescence may occasionally be observed in the perichromosomal region. Target antigens of the AC-6 pattern include Sp100, Sp140/PML and MJ/NXP-2 (2, 8). If a patient is positive for anti-Sp100 and anti-SP140/PML, PBC should be suspected (2, 9, 30). Anti-SP100 exhibits high disease specificity for PBC (8, 9, 30). Additionally, anti-Sp100 antibodies can be observed in other systemic autoimmune rheumatic diseases (such as PSC, AIH, and SLE), though detection rates are all relatively low to varying degrees (9, 32). Anti-MJ/NXP-2 is a common specific antibody associated with juvenile dermatomyositis (JDM) and is also found in adult dermatomyositis (DM) patients (33). When a patient is anti-NXP-2 positive, the possibility of adult DM or JDM can be considered (33, 34).

The few nuclear dots pattern (AC-7) is characterized by a small number of discrete dot-like fluorescent structures (1–6 dots) within the nuclei of interphase cells, corresponding to coiled bodies or Cajal bodies, nuclear gems, etc (2, 8, 35). Cells in mitosis exhibit negative chromosomal staining, and punctate fluorescence may or may not be visible around the chromosomes (2, 8, 35). Target antigens of the AC-7 pattern include p80-coilin (associated with Cajal bodies) and the SMN complex (comprising SMN protein and Gemin proteins) (2, 8, 35, 36). Antibodies against p80-coilin can be detected in asymptomatic individuals, patients with connective tissue diseases (CTD), and those with specific dermatoses (9, 37, 38). However, their positive predictive value for any specific disease is relatively low (9, 37, 38). Anti-SMN antibodies may be observed in mixed connective tissue disease (MCTD), myositis, and other conditions (9, 36, 39).

### 3.3 Nuclear speckled patterns

The fine speckled pattern (AC-4) is characterized by fine speckled fluorescence in the interphase nucleus, with nucleoli either staining positively or being negative (2, 8). During mitosis, the chromosomal regions appear negative for fluorescence, while the nuclear areas outside the chromosomes exhibit granular fluorescence, and the cytoplasm remains unstained (2, 8). Target antigens of the AC-4 pattern include SSB/La, Mi-2, TIF1γ, TIF1β, Ku and TRIM21/Ro52 (2, 8). SSB/La is commonly found in SjD and often coexists with TROVE2/Ro60 (9). In previous guidelines, anti-TROVE2/Ro60 and anti-SSB were both included in the diagnostic criteria for SjD, but in 2016, the ACU/EULAR removed anti-SSB from the SjD diagnostic criteria (40). anti-Mi-2 and anti-TIF1γ are two of the myositis-specific autoantibodies (MSAs) (41–43). anti-Mi-2 detection rates differ between adult DM and JDM (43, 44). anti-TIF1γ and anti-TIF1β are associated with malignancy-related myositis (43, 44). anti-Ku is one of the myositis-associated autoantibodies (MAAs) and can be observed in SSc-AIM (autoimmune myopathy) and SLE-SSc-AIM overlap syndromes (42). If SSB antibodies are further detected in the AC-4 pattern, conditions such as SjD or SLE should be considered (9, 40). Detecting anti-Mi-2, anti-TIF1γ, anti-TIF1β or anti-Ku may suggest the possibility of myositis, and further classification of myositis subtypes can be made based on the specific autoantibodies detected (9, 41–44). Uncertainty frequently surrounds whether anti-TRIM21/Ro52 antibodies yield an AC-4 staining pattern. Although sera displaying an AC-4 pattern may harbour anti-TRIM21/Ro52 antibodies, monospecific anti-TRIM21/Ro52 sera do not yield consistent staining in HEp-2 IFA assays (45).

The myriad discrete speckled nuclear pattern (AC-31), previously named AC-4a (nuclear fine speckled pattern) (46), is characterized by the presence of numerous fine and discrete scattered granules in the nucleoplasm during interphase, presenting the model’s distinctive “multi-discrete-speckled” nuclear morphology (5). Mitotic cells exhibit negative staining for chromosomes, while perichromosomal regions display granular fluorescence with no remarkable staining of the peripheral parts of the cell (5). The AC-31 nuclear pattern is highly associated with anti-TROVE2/Ro60 antibodies (formerly known as anti-TROVE2/Ro60) (5, 47). In contrast, the simple fine speckled pattern (AC-4) shows much weaker correlation with anti-TROVE2/Ro60 antibodies (5, 47). When other antinuclear antibodies are present in positive anti-TROVE2/Ro60 samples, they often obscure the typical features of the AC-31 model, potentially preventing the expected AC-31 pattern from being observed. The AC-31 nuclear pattern is strongly correlated with anti-TROVE2/Ro60 antibodies, and anti-TROVE2/Ro60 is one of the diagnostic criteria for Sjögren Disease (40). Additionally, anti-TROVE2/Ro60 can be found in autoimmune diseases such as SLE but is rarely seen in healthy individuals (5, 9).

The large/coarse speckled pattern (AC-5) is characterized by coarse granular fluorescence in interphase nuclei, with nucleoli either staining positively or remaining negative (2, 8). In mitotic cells, the condensed chromosomal regions show negative fluorescence, while the nuclear areas outside the chromosomes exhibit granular fluorescence, with no cytoplasmic staining (2, 8). The target antigens of the AC-5 pattern include Sm, U1-RNP, hnRNP and RNA polymerase III (2, 8).anti-Sm is one of the diagnostic criteria for SLE, and anti-U1-RNP is included in the diagnostic criteria for MCTD (9, 10, 48). In most cases, the simultaneous detection of AC-5 pattern anti-Sm and anti-U1-RNP may suggest the possibility of SLE, as they often co-occur (9–11). When high-titer anti-U1-RNP is detected alone with negative anti-Sm, MCTD should be considered (9, 48). Anti-Sm is highly specific for SLE whereas anti-U1-RNP frequently occurs in diverse autoimmune diseases (9). The hnRNP complex includes hnRNPA1, A2, B1, B2, C1, C2, etc (49–52). hnRNPA2/B1 is also known as RA33. Anti-RA33 is commonly found in RA and serves as one of its important serum markers (49). Anti-hnRNPA1 and B1 can be observed in SSc (50–52). The functional role of anti-hnRNP C1/C2 in autoimmune diseases remains unclear (53). Anti-RNA polymerase III is closely associated with SSc, particularly in dcSSc (29, 54). Compared to SSc patients positive for topo1 or ACA, those with anti-RNApol-III positivity are more likely to develop renal complications (29, 54).

### 3.4 Nucleolar patterns

The homogeneous nucleolar pattern (AC-8) is characterized by diffuse homogeneous fluorescence staining of the entire nucleolus in interphase cells (2, 8, 54). No staining is observed on the mitotic metaphase plate, while fine granular fluorescence appears at the chromosomal periphery (2, 8, 54). The target antigens of the AC-8 pattern include PM/Scl-75, PM/Scl-100, Th/To, C23/nucleolin, B23/nucleophosmin and NO55/SC65 (nucleolin) (2, 8, 54). When anti-PM/Scl-75, PM/Scl-100 and Th/To are detected in AC-8 pattern, the possibility of SSc may be considered, among which anti- PM/Scl-75 and anti-PM/Scl-100 often appear together (9, 54, 55). Anti-Th/To is predominantly found in patients with limited cutaneous systemic sclerosis (lcSSc) (8, 54). Anti-PM/Scl-75 and anti-PM/Scl-100 can also be detected in polymyositis-systemic sclerosis (PM-SSc) overlap syndrome, DM, PM, and diffuse cutaneous systemic sclerosis (dcSSc) (8, 9, 54–56). The clinical relevance of anti-C23/nucleolin, B23/nucleophosmin, and NO55/SC65 (nucleolin) remains not fully elucidated and requires further evaluation (9, 54).

The clumpy nucleolar pattern (AC-9) is characterized by irregular clumpy staining of the nucleoli and Cajal bodies in interphase cells, as well as perichromosomal staining surrounding the metaphase plate (2, 8, 54). The target antigen of the AC-9 pattern is U3-snoRNP/fibrillarin (2, 8, 54, 57). Autoantibodies against U3-snoRNP/fibrillarin can be detected in patients with SSc, particularly dcSSc, and are occasionally seen in primary Raynaud’s phenomenon and other conditions (9, 54, 57).

The punctate nucleolar pattern (AC-10) is characterized by dense, distinct granular staining confined to the nucleoli of interphase cells (2, 8, 54). Metaphase cells display up to five pairs of bright nucleolar organizer regions (NORs) within the chromatin (2, 8, 54). The cytoplasm of mitotic cells may show mild positive staining. The target antigens of the AC-10 pattern include RNA polymerase I (RNApol-I) and hUBF/NOR-90 (2, 8, 54). By contrast, RNA polymerase II and III are distributed throughout the nucleus, manifesting as the coarse speckled nuclear pattern (AC-5) (54). Since RNApol-I frequently coexists with RNApol-III, attention should be paid to the background AC-5 pattern when interpreting AC-10 results, and accurate distinction requires careful morphological evaluation and professional experience. Given the nearly universal coexistence of RNApol-I and RNApol-III in clinical samples and the current lack of commercially available immunoassays specific for anti-RNAP I antibodies, few studies have differentiated the clinical significance of anti-RNAP I from anti-RNAP III antibodies (9, 54). Accordingly, the established association of anti-RNApol-III with SSc, particularly dcSSc, complicated by renal crisis, is also extrapolated to anti-RNApol-I (9, 54). Anti-hUBF/NOR-90 autoantibodies have been detected in a variety of rheumatic diseases, including SSc, SLE, RA, SjD, and undifferentiated connective tissue disease (UCTD) (9, 54). However, their specific association with distinct diseases or clinical manifestations remains unconfirmed (9, 54). Autoantibodies against RNApol-I, hUBF/NOR-90 and TIF1γ (a target antigen of the AC-4 pattern) are strongly correlated with malignancy] (54, 58).

### 3.5 Nuclear membrane patterns

The smooth nuclear envelope pattern (AC-11) is characterized by homogeneous nuclear staining with intensified fluorescence at the nuclear periphery (2). A distinctive feature is the enhanced fluorescence at the cell-cell contact sites of adjacent cells (2). In metaphase and anaphase cells, the chromosomal plates show no staining. The target antigens of the AC-11 pattern include lamins A, lamins B, lamins C and lamin-associated proteins (LAPs) (2). Lamins A, B, C and LAPs are involved in multiple gene regulatory processes, and their abnormalities are associated with gene mutation-related human diseases, various malignancies, and a spectrum of gastrointestinal disorders (59). Antibodies to Lamins A, B, C and LAPs can be detected in patients with SLE, SS, AIH, as well as in healthy individuals, exhibiting no disease specificity (9, 60, 61).

The punctate nuclear envelope pattern (AC-12) is characterized by punctate staining of the nuclear envelope in interphase cells, with enhanced fluorescence at the contact sites between adjacent cells (2). No staining is observed on the chromatin plates of cells in metaphase and anaphase (2). Target antigens of the AC-12 pattern include nuclear pore complex proteins (gp210, Tpr, p62) and lamin B receptor (LBR) (2). The detection of antibodies to gp210, p62 or LBR in patients supports a suspected diagnosis of PBC, and these antibodies are specific for PBC (9, 30, 60–62). Their positive rates vary, anti-gp210 exhibits a higher prevalence than anti-p62 and LBR, with anti-LBR showing the lowest detection rate (9, 30, 60, 61). Gp210 serves as a specific serological marker for PBC (9, 30, 60, 61). In particular, it is a critical biomarker in patients with AMA-negative PBC (30, 62). By contrast, anti-Tpr can be detected in patients with SLE, SS, AIH and other conditions, yet it lacks disease specificity (2, 9, 60).

### 3.6 Nuclear pleomorphic patterns

The anti-PCNA pattern (AC-13) is characterized by multiple rows of nuclear speckles with variable size and fluorescence intensity. G1 cells show no staining, S phase cells show intense, densely packed speckled, and late S/G2 cells show scattered speckles with occasional nucleoli (2). Condensed chromosomes of mitotic cells are negative for fluorescence. Differentiation of the AC-13 pattern should exclude interference from unclassified ANA patterns (AC-XX). Both the AC-XX-Nuclear SG2NA pattern and AC-XX-Type 1 nuclear pleomorphic pattern also present heterogeneous fluorescence intensity across cells (5, 63, 64). The AC-XX-Nuclear SG2NA pattern shows fine and uniform granular fluorescence in interphase cells, without the scattered speckles observed in late S and early G2 phases of the AC-13 pattern (5, 63, 64). The AC-XX-Type 1 nuclear pleomorphic pattern manifests as large speckled or coarse granular staining in a subset of cells (4, 5). Morphological descriptions and representative images for the AC-XX-Nuclear SG2NA and AC-XX-Type 1 nuclear pleomorphic patterns are available in the International ANA Pattern Consensus (www.ANAPatterns.org) (4, 5). The target antigen of the AC-13 pattern is proliferating cell nuclear antigen (PCNA), which is closely associated with SLE, although its disease specificity remains controversial (2, 9). In addition, PCNA autoantibodies can be detected in other autoimmune diseases such as SSc and RA, as well as in non-autoimmune conditions including hepatitis C virus (HCV) and hepatitis B virus (HBV) infections (2, 9). The clinical relevance of the antibodies to SG2NA protein of the AC-XX-Nuclear SG2NA pattern remains unknown (63, 64), and the target antigen of the AC-XX-Type 1 nuclear pleomorphic pattern is also unidentified (5).

The CENP-F-like pattern (AC-14) is characterized by positive staining of centromeres in prometaphase and metaphase cells, manifesting as numerous regularly arranged, small, and faint fluorescent dots (2). Interphase nuclei exhibit granular fluorescence of variable intensity, with the strongest staining in the G2 phase and the weakest or absent staining in the G1 phase (2). Most prometaphase cells show faint nuclear envelope staining. In addition, some serum samples display intense staining of the midbody ring (stem body) at the cleavage furrow of daughter cells during anaphase and telophase. Diffuse cytoplasmic staining can be observed in mitotic cells. The target antigen of the AC-14 pattern is centromere protein F (CENP-F) (2). Anti-CENP-F antibodies have been identified in various malignancies, autoimmune diseases and neurological disorders and their clinical significance remains to be further clarified (9, 65). Close clinical surveillance is recommended for elderly patients with gradually elevated antibody titers, as this may indicate an increased risk of malignancy (65).

## 4 Cytoplasmic patterns of HEp-2 IFA: morphological features, immunological targets and clinical relevance

Cytoplasmic patterns on HEp-2 IFA are classified by morphological similarity into three categories: cytoskeletal patterns, including fibrillar linear/actin-like (AC-15), fibrillar filamentous (AC-16) and fibrillar segmental/vinculin-like (AC-17), cytoplasmic speckled patterns, including cytoplasmic discrete dots/GW body-like (AC-18), cytoplasmic dense speckled (AC-19) and cytoplasmic fine speckled (AC-20), and other cytoplasmic patterns, including cytoplasmic reticular/AMA (AC-21), cytoplasmic polar/Golgi-like (AC-22) and cytoplasmic rods and rings (AC-23) (2). The morphological characteristics, target antigens and clinical relevance of cytoplasmic patterns are summarized in **Table 2**.

**Table 2:** The morphological characteristics, target antigens and clinical relevance of cytoplasmic patterns.

Cytoplasmic patterns		Morphological characteristics	Antigens	Disease association
Cytoskeletal patterns	AC-15	The staining of cytoskeletal filaments	Actin	AIH-1(F-actin)
			Non-muscle myosin	Potential therapeutic target
	AC-16	The staining of microtubules and intermediate filaments radiating outward from the perinuclear region	Keratin	RA, Pulmonary fibrosis
			Tubulin, vimentin, tropomyosin	rare in SARD
	AC-17	The intensified staining along short segments of stress fibers and periodic dense bodies	α-actinin	SLE, LN, AIH-1
			Vinculin	Irritable Bowel Syndrome (IBS)
Cytoplasmic speckled patterns	AC-18	The discrete punctate fluorescence distributed throughout the cytoplasm	GW/P bodies (GW182, Su/Ago2, Ge-1/HEDLS/EDC4), endosomes (EEA1, CLIP-170, GRASP-1, LBPA), lysosomes, diacyl-phosphatidylethanolamine, processing bodies	SARDs, neurological disorders, cancers, other conditions
	AC-19	An almost homogeneous, hazy, indistinct fluorescent appearance throughout the entire cytoplasm	RibP	SLE (NPSLE, LN, SLE-related hepatitis)
			tRNA-synthetases (PL-7, PL-12)	Myositis-specific antibodies (MSA), AIM, particularly ASS
			SRP	AIM, particularly IMNM
	AC-20	The scattered fine punctate staining in the cytoplasm	Jo-1	MSA, PM/DM, ASS
Other cytoplasmic patterns	AC-21	The coarse granular reticular staining in the cytoplasm	PDC-E2/M2, BCOADC-E2, OGDC-E2, E1α subunit of PDC and E3BP/protein X	PBC (high specificity), SSc, PBC-SSc, PBC-SjD overlap, other diseases
	AC-22	The discontinuous punctate or granular ribbon-like staining distributed polarly on one side of the nucleus	Golgin complex (giantin/macrogolgin, golgin-97, golgin-95/GM130, golgin-160, golgin-245)	Low disease positive predictive value for SARDs
	AC-23	The distinct or small rod-shaped and ring-shaped structures in the cytoplasm of interphase cells	IMPDH2	Common in HCV interferon or ribavirin-treated patients, rare in autoimmune diseases and other conditions

AIH-1, 1 type autoimmune hepatitis; RA:rheumatoid arthritis; SLE, systemic lupus erythematosus; LN, lupus nephritis; NPSLE, neuropsychiatric SLE; AIM, autoimmune myopathies; ASS, antisynthetase syndrome; DM, dermatomyositis; PM:polymyositis; PBC, primary biliary cholangitis; SARDs, systemic autoimmune rheumatic diseases; SSc systemic sclerosis; HCV, hepatitis C virus.

### 4.1 Fibrillar/cytoskeletal patterns

The cytoplasmic fibrillar linear/actin-like pattern (AC-15) is characterized by staining of cytoskeletal filaments (2). The typical appearance shows striated actin bundles running along the long axis of the cell, occasionally presenting as fine discontinuous granular deposits (2). The target antigens of AC-15 are actin and non-muscle myosin (1, 2, 9). Actin exists as F-actin and G-actin (2, 9). Antibodies to F-actin exhibits stronger clinical relevance to autoimmune diseases than G-actin (2, 9). F-actin serves as the major target antigen of anti-smooth muscle antibodies, which are serological markers specific for type 1 autoimmune hepatitis (AIH-1), and correlates with AIH-1 disease activity (1, 9, 66, 67). In addition, F-actin autoantibodies can be detected in PBC, celiac disease, and chronic active hepatitis (1, 9, 62). Among non-muscle myosins, non-muscle myosin II (NM II) is the most extensively studied and is associated with malignancies, psychiatric disorders, inflammation and other related conditions (68, 69). Non-muscle myosin II represents a promising therapeutic target (69). A myosin II inhibitor for the treatment of obstructive hypertrophic cardiomyopathy has been approved by the FDA (69).

The cytoplasmic fibrillar filamentous pattern (AC-16) is characterized by staining of microtubules and intermediate filaments radiating outward from the perinuclear region (2). Target antigens of AC-16 include tubulin, vimentin, cytokeratin and tropomyosin (2). Due to the diversity of corresponding antigens, the AC-16 pattern manifests with variable immunofluorescence appearances. Currently, no commercial specific immunoassays are available for the detection of tubulin, vimentin or tropomyosin (1, 9). These autoantibodies can be identified in a wide spectrum of diseases and are generally absent in systemic autoimmune rheumatic diseases (SARDs), while their clinical relevance remains to be further clarified (1, 9). Tropomyosin autoantibodies may serve as a biomarker for ulcerative colitis and help distinguish it from Crohn’s disease (70). Anti-keratin antibodies demonstrate relatively high specificity for RA, correlate with disease activity, and can be used as an auxiliary serological indicator for RA diagnosis (71). Keratin 8/18/19 autoantibodies are detectable in autoimmune diseases such as AIH, and keratin 18/19 may also contribute to the progression of pulmonary fibrosis (9, 72–74).

The cytoplasmic fibrillar segmental/vinculin-like pattern (AC-17) shows intensified staining along short segments of stress fibers and periodic dense bodies (2). The target antigens of AC-17 are α-actinin and vinculin (2). α-actinin exhibits cross-reactivity with dsDNA and is significantly correlated with disease activity in SLE, particularly lupus nephritis (LN) (75–77). Furthermore, anti-α-actinin antibodies may act as a novel predictive biomarker for therapeutic response in AIH-1 [72] (78). Dysregulation of vinculin has been implicated in multiple disorders, including cardiovascular diseases, gastrointestinal diseases and malignancies (79). Anti-vinculin antibodies are regarded as one of the potential biomarkers for irritable bowel syndrome (IBS), though related research is still in its preliminary stage (1, 80). At present, no commercial specific immunoassays are available for α-actinin and vinculin (1, 9). Although these autoantibodies can be detected in various diseases, their clinical significance remains to be fully elucidated (9).

### 4.2 Cytoplasmic speckled patterns

The cytoplasmic discrete dots/GW body-like pattern (AC-18) is characterized by discrete punctate fluorescence distributed throughout the cytoplasm (2). Target antigens of the AC-18 pattern include GW/P bodies (GW182, Su/Ago2, Ge-1/HEDLS/EDC4), endosomes (EEA1, CLIP-170, GRASP-1, LBPA), lysosomes,diacyl-phosphatidylethanolamine and processing bodies (1, 2, 81). Autoantibodies corresponding to the AC-18 pattern have been detected in various SARDs and other disorders (9). However, its prevalence and clinical associations have been inconsistently reported across studies, and remain poorly defined in broader or disease-specific patient cohorts (9). For instance, several studies have indicated that the AC-18 pattern is most frequently observed in SjD, SLE and neurological disorders, such as ataxia and mixed motor-sensory neuropathy (1, 9). A Taiwanese study demonstrated that individuals positive for the AC-18 pattern had significantly higher prevalence rates of malignancy and autoimmune diseases compared with ANA-negative subjects (82). Additional studies have also linked the AC-18 pattern to PBC, RA, AIM and multiple types of cancers (83). The specificity and diagnostic value of these autoantibodies in the above diseases have not been fully established.

The cytoplasmic dense speckled pattern (AC-19) is defined by an almost homogeneous, hazy, indistinct fluorescent appearance throughout the entire cytoplasm (2). The AC-19 pattern needs to be differentiated from the fine punctate fluorescence seen in rare clustered cells of the AC-XX-cytoplasmic variable-intensity dense speckled pattern (84). Morphological descriptions and representative images for the AC-XX-cytoplasmic variable-intensity dense speckled patterns are available in the International ANA Pattern Consensus (www.ANAPatterns.org). Target antigens of the AC-19 pattern include ribosomal P proteins (RibP), tRNA synthetases (PL-7, PL-12) and the signal recognition particle (SRP) (2). Anti-RibP antibodies are highly specific for SLE and represent potential serological biomarkers (1). Detection of anti-RibP antibodies in sera with the AC-19 pattern supports a probable diagnosis of SLE (9). Anti-RibP antibodies may also predict disease exacerbation, involving neuropsychiatric SLE (NPSLE), lupus nephritis (LN) and SLE-related hepatitis (9). PL-7 and PL-12 are two well-recognized MSAs. Identification of anti–PL-7 or anti–PL-12 antibodies in AC-19-positive samples suggests AIM, particularly antisynthetase syndrome (ASS) (9, 85). Seropositivity for PL-7 or PL-12 is associated with a higher likelihood of ASS-related manifestations, including Raynaud’s phenomenon and interstitial lung disease (ILD) (85). When anti-SRP antibodies are detected in the AC-19 pattern, a diagnosis of AIM, especially immune-mediated necrotizing myopathy (IMNM), should be considered (9, 86). Anti-SRP antibodies have already been included in the relevant diagnostic criteria (86).

The cytoplasmic fine speckled pattern (AC-20) manifests as scattered fine punctate staining in the cytoplasm, generally with a diffusely homogeneous or densely fine speckled background (2). The target antigen of the AC-20 pattern is Jo-1/histidyl-tRNA synthetase (2). As one of the myositis-specific autoantibodies (MSAs), Jo-1 serves as a specific diagnostic biomarker for polymyositis/dermatomyositis (PM/DM) and has been incorporated into the AIM classification criteria (9). Detection of anti–Jo-1 antibodies raises suspicion of AIM, particularly antisynthetase syndrome (ASS) and overlap syndromes such as PM/DM-CTD and PM/DM-MCTD (9). Patients with positive anti–Jo-1 antibodies are more prone to presenting with Raynaud’s phenomenon, polyarthritis, interstitial lung disease (ILD) and mechanic’s hands (9). Notably, the AC-20 pattern itself is not specific for Jo-1 (9). Therefore, AIM cannot be suspected solely based on the presence of the AC-20 pattern (9). Further confirmation of positive Jo-1 serology is required to support a potential diagnosis of AIM (9).

### 4.3 Other cytoplasmic patterns

The cytoplasmic reticular/AMA pattern (AC-21) is characterized by coarse granular reticular staining in the cytoplasm, extending from the nuclear envelope throughout the entire cytoplasm (2). The AC-21 pattern must be differentiated from the cell cycle-dependent coarse reticular granular fluorescence caused by the AC-XX-cytoplasmic variable-intensity coarse reticular pattern, in which not all cells exhibit coarse fibrillar staining (5). A typical representative image and the description of the AC-XX-cytoplasmic variable-intensity coarse reticular pattern are available in the International Consensus on ANA Patterns (www.ANAPatterns.org) (4, 5). Additionally, AC-21 should be distinguished from the AC-XX-cytoplasmic fine reticular pattern, which exhibits fine reticular staining throughout the cytoplasm (antigen unknown) (5). Sera positive for AMA-M2 display the classic AC-21 pattern. AC-21 pattern targets members of the mitochondrial 2-oxoacid dehydrogenase complex (2-OADC) family, including PDC-E2/M2, BCOADC-E2, OGDC-E2, the E1α subunit of PDC and E3BP/protein X (2, 87). AMA-M2 is one of the diagnostic criteria and a specific serological marker for PBC (9, 30). It is also detectable in other autoimmune diseases such as SSc, PBC-SSc overlap syndrome and PBC-SjD overlap syndrome (9).

The cytoplasmic polar/Golgi-like pattern (AC-22) is characterized by discontinuous punctate or granular ribbon-like staining around the nucleus, distributed polarly on one side of the nucleus (2). The target antigens are Golgi apparatus proteins, mainly giantin/macrogolgin, golgin-97, golgin-95/GM130, golgin-160 and golgin-245 (2, 88). The AC-22 pattern is occasionally seen in some autoimmune diseases (SLE, RA, inflammatory myopathies, Crohn’s disease, SSc, etc.) and non-autoimmune diseases (thyroid disorders, viral infections, cancers, etc.), lacking disease specificity and having low diagnostic value for SARDs (2, 9, 88).

The cytoplasmic rods and rings pattern (AC-23) is characterized by distinct or small rod-shaped and ring-shaped structures in the cytoplasm of interphase cells (2). The target antigen is inosine monophosphate dehydrogenase 2 (IMPDH2) (2). It is commonly observed in HCV patients treated with interferon or ribavirin (2, 9, 89–91). Although anti-rods and rings (anti-RR) antibodies are rare and lack disease specificity, they have been reported in a wide spectrum of conditions: autoimmune diseases (RA, AIH, SS, ankylosing spondylitis), renal diseases (renal insufficiency, renal failure), liver diseases (HBV, HCV), pulmonary diseases (pulmonary embolism, interstitial lung disease), HIV, arthropathies, users of immunosuppressive drugs and even healthy individuals (2, 9, 89–91).

## 5 Mitotic patterns of HEp-2 IFA: morphological features, immunological targets and clinical relevance

Mitotic patterns include AC-24 to AC-28 patterns. Their fluorescence patterns differ significantly, making them relatively easy to distinguish. The morphological characteristics, target antigens and clinical relevance of mitotic patterns are summarized in **Table 3**.

**Table 3:** The morphological characteristics, target antigens and clinical relevance of mitotic patterns.

Mitotic patterns	Morphological characteristics	Antigens	Disease association
AC-24	The distinct centrioles/centrosomes (1–2 per cell) distributed in the cytoplasm and at both poles of the mitotic spindle	Centrosome (pericentrin, ninein, Cep110, Cep250, enolase (α, γ), PCM-1, PCM-2, Mob1)	Low disease positive predictive value
AC-25	The conical staining of spindle fibers in mitotic cells	HsEg5/KIF11/NuMA2	Low disease positive predictive value
AC-26	The speckled fluorescence of spindle fibers and nuclei in mitotic cells	NuMA1/centrophilin/SP-H/NMP-22	SARDs
AC-27	The positive staining of the intercellular bridge between two daughter cells during late mitosis	Aurora kinase B, INCENP, MPP1/KIF2B, KIF-14, MKLP-1, CENP-E, CENP-F, TD60, MSA36	Low disease positive predictive value
AC-28	The punctate staining of chromosomes in prophase and metaphase cells	Chromosome coat protein, DCA, MCA-1, MCA-5	Low disease positive predictive value

SARDs, systemic autoimmune rheumatic diseases.

The centrosome pattern (AC-24) is characterized by distinct centrioles/centrosomes (1–2 per cell) distributed in the cytoplasm and at both poles of the mitotic spindle (2). Target antigens of AC-24 are centrosome-associated proteins, including PCM-1, PCM-2, pericentrin (PCNT), ninein, Cep110, Cep250, α-enolase, γ-enolase and Mob1 (2). The AC-24 pattern is extremely rare and occasionally detected in patients with various autoimmune and non-autoimmune diseases, such as SSc, SjD, RA, PBC, SLE, Raynaud’s syndrome, infections, calcification disorders and pulmonary hypertension (1, 2, 9, 27). However, it has a low positive predictive value for any specific disease (1, 9).

The spindle fibers/non-NuMA pattern (AC-25) is characterized by conical staining of spindle fibers in mitotic cells, whereas interphase cells show no staining (2). The target antigen of AC-25 is the spindle motor protein Eg5 (HsEg5/KIF11/NuMA2) (2, 92). AC-25 is exceedingly rare in routine serological testing. It can be detected in SS, SLE and other systemic autoimmune rheumatic diseases, but exhibits low positive predictive value for disease diagnosis (1, 2, 9, 27).

The NuMA-like pattern (AC-26) is characterized by speckled fluorescence of spindle fibers and nuclei in mitotic cells, accompanied by prominent nuclear punctate staining. Interphase cells display homogeneous fine granular fluorescence (2, 46). Target antigens of AC-26 include nuclear mitotic apparatus protein 1 (NuMA1)/centrophilin/spindle pole–nucleus (SP-H)/nuclear matrix protein 22 (NMP-22) (2, 93). AC-26 is rarely encountered in routine serological practice. Approximately half of positive cases are associated with autoimmune rheumatic diseases such as SLE, SS and UCTD, while it is seldom seen in non-autoimmune conditions (1, 2, 9, 27). Notably, at high ANA titers, up to 40% of AC-26-positive cases are correlated with connective tissue diseases (CTDs) (1). The presence of anti-mitotic spindle apparatus antibodies may serve as a unique serological marker for CTDs (1).

The intercellular bridge pattern (AC-27) is characterized by positive staining of the intercellular bridge between two daughter cells during late mitosis, which disappears once cell division is completed. Interphase cells are generally negative (2). Putative target antigens of AC-27 include Aurora kinase B, INCENP, MPP1/KIF2B, kinesin-like protein 14 (KIF-14), mitotic kinesin-like protein 1 (MKLP-1), CENP-E, CENP-F, TD60 and MSA36 (1, 2). This pattern is extremely rare in routine serological detection and has been occasionally observed in systemic sclerosis, Raynaud’s phenomenon, and malignancies, with a very low disease positive predictive value (1, 2, 9, 27).

The mitotic chromosomal pattern (AC-28) is characterized by punctate staining of chromosomes in prophase and metaphase cells, while interphase cells are unstained (2). Target antigens of AC-28 include chromosome coat protein, dividing cell antigen (DCA), mitotic chromosome autoantigen 1 (MCA-1) and MCA-5 (2). AC-28 is extremely rare in routine laboratory practice and has been sporadically reported in SLE, discoid lupus erythematosus, chronic lymphocytic leukemia, SjD, polymyalgia rheumatica, and various tumors, with very low positive predictive value for specific diseases (1, 2, 9, 27).

## 6 Discussion

The target antigens of the AC-1, AC-2, AC-29 and AC-30 patterns are all well characterized but distinct from one another, with substantially different clinical relevance. Therefore, accurate differentiation among these patterns is clinically critical. Similarly, the AC-13 pattern resembles the AC-XX-Nuclear SG2NA and AC-XX-Type 1 nuclear pleomorphic patterns (5, 63, 64). However, the clinical relevance of the SG2NA protein (target antigen of AC-XX-Nuclear SG2NA) remains unclear, and the target antigen of AC-XX-Type 1 nuclear pleomorphic pattern is still unidentified (5, 63, 64). Precise discrimination between these patterns is also essential. Analogous challenges exist in distinguishing AC-19 from AC-XX-cytoplasmic variable-intensity dense speckled pattern, as well as differentiating AC-21 from AC-XX-cytoplasmic variable-intensity coarse reticular pattern and AC-XX-cytoplasmic fine reticular pattern. The nuclear dense fine speckled pattern (AC-2) is frequently detected in healthy individuals or patients with non-autoimmune diseases (2, 5, 7, 9, 20). It has strong implications for the exclusion of systemic autoimmune rheumatic diseases (SARDs). Accordingly, it is vital to reliably distinguish the nuclear dense fine speckled pattern (AC-2) from other nuclear patterns with stained metaphase chromosome plates, particularly the fine-speckled with mitotic plate pattern (AC-30). The addition of the newly defined AC-30, AC-31 and partial AC-XX unclassified patterns underscores the need to systematically summarize the morphological characteristics, immunological features and clinical relevance of ANA indirect immunofluorescence patterns. Such a summary helps to further elucidate the discrepant clinical associations among morphologically similar fluorescence patterns. For this reason, we classified and compared AC-1 to AC-31 according to their morphological similarities.

Furthermore, there remains no systematic overview regarding the similarities and differences in clinical relevance among different target antigens within the same ANA pattern. For instance, positivity for antibodies to the classic target antigens of AC-1 (dsDNA, histones and nucleosomes) alerts clinicians to a potential diagnosis of SLE and DIL, whereas unidentified antigens in the same pattern may be associated with RA, AIH, juvenile idiopathic arthritis and other conditions (2, 7, 9). Detailed differential interpretations of clinical relevance are also required for AC-30, AC-6, AC-4, AC-5, AC-8 and AC-10 patterns. Accordingly, we summarized the consensus and discrepancies in clinical relevance among different autoantibodies within each pattern. This facilitates an in-depth understanding of distinct clinical correlations of different target antigens under the same fluorescence pattern, aids laboratory analysts and clinicians in pattern recognition and interpretation, and further optimizes the clinical application of HEp-2 IFA in the diagnostic workup of patients with suspected autoimmune diseases.

This review focuses on the morphological and immunological characteristics of ANA patterns detected by indirect immunofluorescence, as well as their clinical associations with autoimmune diseases. We did not systematically elaborate the molecular composition, functional mechanisms of various target antigens, or their relationships with non-autoimmune diseases. Therefore, this review has limited guiding value for basic mechanistic research on target antigens and their correlations with non-autoimmune disorders. In addition, experimental detection methods for individual target antigens were not comprehensively discussed and were only briefly outlined in the section “2. Methods for ANA detection”, which limits the practical laboratory application of this review. Differences in autoantibody prevalence related to gender, ethnicity and age are generally recognized epidemiological features and thus were not repeatedly described for each individual pattern (94).

Current studies on automated immunofluorescence interpretation mainly focus on AI models based on cellular morphology for ANA, ANCA and dsDNA detection. Among these, AI algorithms for ANA interpretation have been most extensively investigated, and several mature models have been applied in clinical practice. Nevertheless, most clinically available ANA-AI models can only recognize common basic patterns, such as homogeneous nuclear, speckled nuclear, nucleolar, centromere and nuclear dot patterns. At present, there are no well-established mature models capable of distinguishing subtle subtypes such as nuclear fine speckled and nuclear coarse speckled patterns, nor any intelligent recognition systems covering the full spectrum of AC-1 to AC-31 patterns. Considering the inherent difficulty of manual interpretation in HEp-2 IFA, this review systematically compares and differentiates morphologically similar fluorescence patterns. We anticipate that this work will assist fluorescence interpreters in accurately identifying ANA patterns, provide reliable evidence for clinical decision-making, and facilitate the development of full-spectrum ANA-AI automatic interpretation models.

Some clinical studies have enrolled relatively small cohorts, and their conclusions require further validation. For example, the negative correlation of anti-histone and anti-nucleosome antibodies with pulmonary fibrosis as a complication of SSc and MCTD was derived from a cohort of only 121 subjects, whether this negative association can be generalized to larger populations remains debatable. Similar limitations exist in clinical correlation studies of target antigens of other ANA patterns. In this review, we primarily focused on diseases with robust evidence of antigen association and did not list all relevant disorders for every target antigen. In analyzing the clinical relevance of patterns such as nuclear dense fine speckled pattern (AC-2), we only included studies published after the 2015 ICAP standardization.

In routine laboratory practice, low-titer ANA positivity is frequently observed in healthy adults from different geographical regions. We recommend that individual laboratories and hospitals adjust their local ANA cutoff titers according to regional population characteristics. In addition, ANA titers tend to increase with age to varying degrees. Hence, separate reference cutoffs should be established for children and adults rather than adopting a uniform standard.

With the continuous identification of novel ANA target antigens, the clinical implications of known fluorescence patterns are continually expanding. At present, commercial specific immunoassays are unavailable for a considerable number of autoantigens, including the SMN complex (AC-7), C23/nucleolin, B23/nucleophosmin, NO55/SC65 (AC-9) and lamins A, B, C (AC-11), Tpr, p62 and LBR (AC-12), CENP-F (AC-14), tubulin, vimentin and tropomyosin (AC-16), α-actinin and vinculin (AC-17), GW/P bodies, endosomes, lysosomes and processing bodies (AC-18), golgin complex (AC-22), IMPDH2 (AC-23), centrosome proteins (AC-24), HsEg5 (AC-25), NuMA1/SP-H/NMP-22 (AC-26), Aurora kinase B, INCENP, MPP1/KIF2B, KIF-14, MKLP-1, CENP-E, CENP-F, TD60 and MSA36 (AC-27), as well as chromosome coat protein, DCA, MCA-1 and MCA-5 (AC-28). These limitations hinder the exploration of their biological functions and clinical relevance. Furthermore, many studies only report disease associations of autoantibodies against these antigens, lacking direct correlation analysis with the corresponding HEp-2 IFA patterns. Other studies describe the clinical relevance of IFA patterns without clearly defining their underlying target antigens, thereby limiting the clinical applicability of these findings.

Some target antigens yield highly similar fluorescence patterns. For example, no commercial specific immunoassays are available for AC-18-related antigens, and the staining patterns induced by GW bodies are morphologically indistinguishable from those caused by endosomes and lysosomes under light microscopy. Differentiation can only be achieved using research-grade reagents, leaving their clinical relevance insufficiently validated. The increasing number of unclassified AC-XX patterns reflects the continuous discovery of unknown target antigens and the expanding diversity of ANA fluorescence patterns. The clinical significance of many ANA patterns and their corresponding target antigens remains to be further explored.

With the identification of increasingly novel patterns and unknown target antigens, ANA research continues to advance. Nevertheless, a comprehensive and thorough understanding of the full spectrum of ANA profiles remains a long-term endeavor. In the detection of other autoantibodies, quantitative assays are gradually replacing qualitative methods. However, for ANA testing, HEp-2 IFA will remain irreplaceable for a long time. Further in-depth research on HEp-2 IFA patterns is still urgently needed.
